# Age-Associated Changes in the Vascular Renin-Angiotensin System in Mice

**DOI:** 10.1155/2016/6731093

**Published:** 2016-04-20

**Authors:** Hye Eun Yoon, Eun Nim Kim, Min Young Kim, Ji Hee Lim, In-Ae Jang, Tae Hyun Ban, Seok Joon Shin, Cheol Whee Park, Yoon Sik Chang, Bum Soon Choi

**Affiliations:** ^1^Division of Nephrology, Department of Internal Medicine, College of Medicine, The Catholic University of Korea, Seoul 137701, Republic of Korea; ^2^Department of Internal Medicine, Incheon St. Mary's Hospital, Incheon, Republic of Korea; ^3^Department of Internal Medicine, Seoul St. Mary's Hospital, Seoul, Republic of Korea; ^4^Department of Internal Medicine, Yeouido St. Mary's Hospital, Seoul, Republic of Korea

## Abstract

*Background*. This study evaluated whether the change in the renin-angiotensin system (RAS) is associated with arterial aging in mice.* Methods*. Histologic changes and expressions of transforming growth factor-*β* (TGF-*β*), collagen IV,* fibronectin*, angiotensin II (Ang II), angiotensin-converting enzyme (ACE), angiotensin-converting enzyme 2 (ACE2), angiotensin II type 1 receptor (AT1R), angiotensin II type 2 receptor (AT2R), prorenin receptor (PRR), Mas receptor (MasR), endothelial nitric oxide synthase (eNOS), NADPH oxidase 2 and oxidase 4 (Nox2 and Nox4), 8-hydroxy-2′-deoxyguanosine (8-OHdG),* 3-nitrotyrosine*, and superoxide dismutase 1 and dismutase 2 (SOD1 and SOD2) were measured in the thoracic aortas from 2-month-old, 12-month-old, and 24-month-old C57/BL6 mice.* Results*. Twenty-four-month-old mice showed significantly increased aortic media thickness and expressions of TGF-*β*, collagen IV, and fibronectin, compared to 2-month-old and 12-month-old mice. The expressions of PRR, ACE, and Ang II, and AT1R-positive area significantly increased, whereas expressions of ACE2 and MasR and AT2R-positive area decreased with age. The expressions of phosphorylated serine^1177^-eNOS, SOD1, and SOD2 decreased, and the 8-OHdG-positive area and the 3-nitrotyrosine-positive area increased with age. The expression of Nox2 significantly increased with age, but that of Nox4 did not change.* Conclusions*. The enhanced PRR-ACE-Ang II-AT1R axis and reduced ACE2-MasR axis were associated with arterial aging in mice.

## 1. Introduction

Aging is the major risk factor of vascular disease and vascular structural and functional changes associated with aging are implicated in the increased cardiovascular risk of the elderly population [1]. Arterial aging is characterized by increased luminal diameter with wall remodeling, intimal and medial thickening, reorganization of the extracellular matrix, vascular stiffening, and endothelial dysfunction [2]. Various mechanisms are involved in the arterial aging process, including mitochondrial dysfunction, oxidative stress, altered calcium regulation, increased DNA protein and lipid oxidation, inflammation, and the activation of the renin-angiotensin-system (RAS) [3]. Additionally, changes in the activity or responsiveness of the RAS occur with aging [4].

Angiotensin (Ang) II is the main effector peptide in the RAS-associated vascular aging [5]. Ang II acts through two distinct receptor subtypes, Ang II type 1 receptor (AT1R) and Ang II type 2 receptor (AT2R), which have counterregulatory interactions in the cardiovascular and renal system [6]. Activation of the AT1R promotes vasoconstriction, reactive oxygen species (ROS) production, extracellular matrix remodeling, and inflammation [7]. On the other hand, the AT2R inhibits cell growth, inflammation, and fibrosis and exerts cardioprotective effects against ischemia-reperfusion injury and acute myocardial infarction [8, 9]. The angiotensin-converting enzyme (ACE) converts Ang I to Ang II, whereas the angiotensin-converting enzyme 2 (ACE2) metabolizes Ang II and generates Ang-(1-7) [10]. Ang-(1-7) does not activate AT1R or ATR2 but acts at the G-protein-coupled Mas receptor (MasR) [11]. The ACE2-Ang-(1-7)-MasR axis exerts vasodilator, antiproliferative, and antifibrotic actions opposed to those of the ACE-Ang II-AT1R axis [12]. Besides these components, there is prorenin receptor (PRR) which binds renin as well as its precursor, prorenin. The binding of prorenin or renin to PRR activates the intracellular signaling pathway and allows for the conversion of angiotensinogen to Ang I and Ang I to Ang II by the prorenin/PRR complex [13–15].

There is little data regarding the change in RAS in aorta of aging mice. As the activation of RAS is closely linked with aging process and cardiovascular disease, we hypothesized that the aorta of aging mice may exhibit altered expression of PRR-ACE-Ang II-AT1R axis and ACE2-MasR axis. This study evaluated the expression of RAS components and associated proinflammatory, profibrotic, and antioxidant molecular changes in thoracic aorta tissues of aging mice.

## 2. Materials and Methods

### 2.1. Animal Model

The Animal Care Committee of the Catholic University approved the experimental protocol. Aging C57BL/6 mice were purchased from the Korea Research Institute of Bioscience and Biotechnology (Chungcheongbuk-do, Republic of Korea). Mice were housed in a controlled temperature and controlled light environment. Mice were divided into three groups as follows: 2-month-old group (2 M group, *n* = 6), 12-month-old group (12 M group, *n* = 6), and 24-month-old group (24 M group, *n* = 6).

### 2.2. Histology and Microscopic Analysis

Thoracic aorta tissues were embedded in low-temperature melting paraffin, and 4 *μ*m sections were processed and stained with hematoxylin-eosin (HE). HE staining was analyzed with a color-image analyzer (TDI Scope Eye, Version 3.5 for Windows, Olympus, Tokyo, Japan). The media thickness was assessed by measuring the length of the media at 10 different positions in six different sections per animal.

### 2.3. Immunohistochemistry

Deparaffinized tissue sections were processed for immunohistochemistry as described elsewhere [16] using primary antibodies to AT1R (Santa Cruz Biotechnology, TX, USA), AT2R (Novus Biologicals, CO, USA), MasR (Novus Biologicals, CO, USA), 8-hydroxy-2′-deoxyguanosine (8-OHdG, Japan Institute for the Control of Aging, Shizuoka, Japan), and 3-nitrotyrosine (Santa Cruz Biotechnology, TX, USA). All sections were assessed using a color-image analyzer (TDI Scope Eye, Version 3.5 for Windows, Olympus).

### 2.4. Western Blot Analysis

Total protein was extracted from the thoracic aorta tissues with a Pro-Prep Protein Extraction Solution (Intron Biotechnology, Gyeonggi-do, Republic of Korea) according to the manufacturer's instructions. Western blot analysis was performed using the following antibodies: transforming growth factor-*β* (TGF-*β*, R&D Systems, MN, USA), collagen IV (Abcam, Cambridge, UK), fibronectin (Proteintech Group Inc., IL, USA), Ang II (Novus Biologicals, CO, USA), ACE (Santa Cruz Biotechnology, TX, USA), ACE2 (R&D Systems, MN, USA), AT1R (Santa Cruz Biotechnology, TX, USA), AT2R (Novus Biologicals, CO, USA), PRR (Sigma Life Science, MO, USA), MasR (Novus Biologicals, CO, USA), endothelial nitric oxide synthase (eNOS, Cell Signaling Technology Inc., MA, USA), NADPH oxidase 2 (Nox2, BD Biosciences, MD, USA) and NADPH oxidase 4 (Nox4, Santa Cruz Biotechnology, TX, USA), superoxide dismutase 1 (SOD1, Enzo Life Sciences, NY, USA), superoxide dismutase 2 (SOD2, Abcam, Cambridge, UK), and *β*-actin (Sigma Life Science, MO, USA).

### 2.5. Enzyme Immunoassay

Serum levels of renin (Cloud-Clone Corp., TX, USA) and Ang II (Lifespan biosciences, WA, USA) were measured using enzyme-linked immunosorbent assay and competitive enzyme immunoassay, respectively, according to the assay protocols.

### 2.6. Statistical Analysis

Data are expressed as means ± standard deviation (SD). Differences between the groups were examined for statistical significance using ANOVA with Bonferroni correction (SPSS). A *P* value of less than 0.05 was considered statistically significant.

## 3. Results

### 3.1. Influence of Age on Thoracic Aorta Media Thickness and Expressions of TGF-*β*, Collagen IV, and Fibronectin

**Figure 1:**
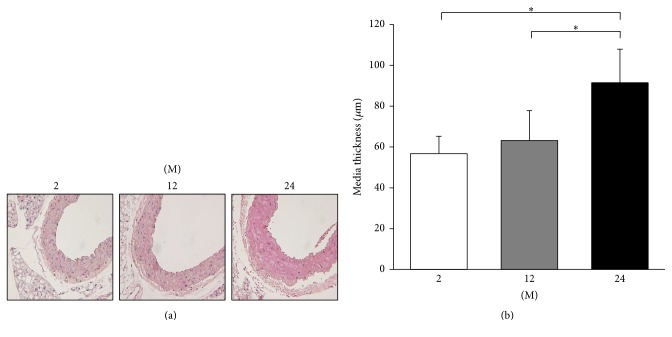
Thoracic aorta media thickness. (a) Representative images of HE stain of thoracic aorta tissues (original magnification, 400x). (b) The 24 M group (91.47 ± 16.41 *μ*m) showed significantly increased aortic media thickness compared to 2 M (56.74 ± 8.51 *μ*m) and 12 M groups (63.19 ± 14.67 *μ*m). ^*∗*^ *P* < 0.001. Values are shown as mean ± SD.

**Figure 2:**
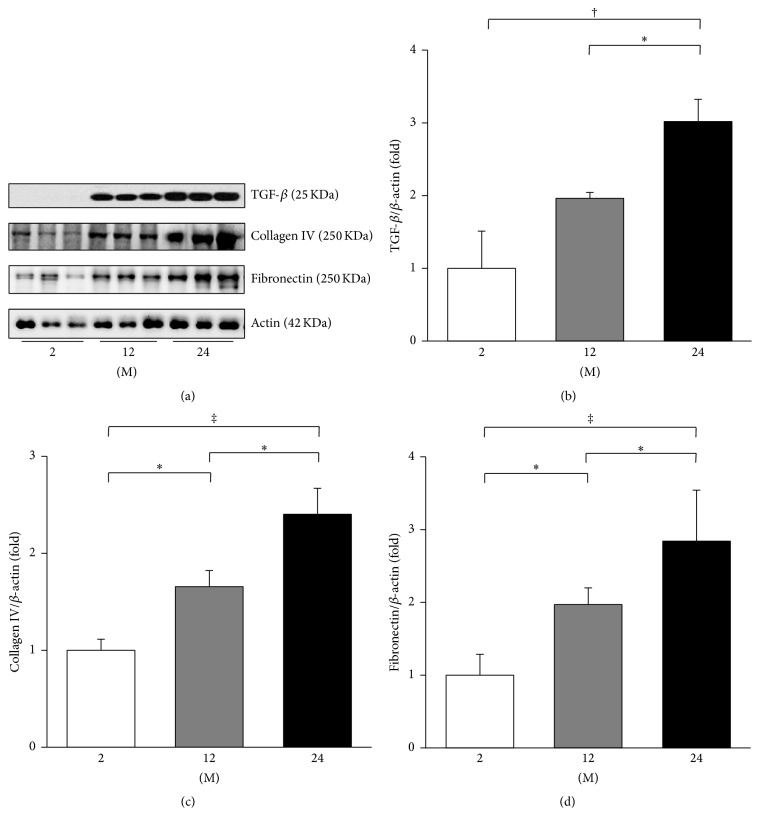
Expressions of TGF-*β*, collagen IV, and fibronectin. (a) Representative western blots of TGF-*β*, collagen IV, and fibronectin. (b) The expression of TGF-*β* significantly increased in the 24 M group compared with the 2 M and 12 M groups. (c) The expression of collagen IV gradually increased with age. (d) The expression of fibronectin significantly increased with age. ^*∗*^ *P* < 0.05, ^†^ *P* < 0.01, and ^‡^ *P* < 0.001. Values are shown as mean ± SD.

The media thickness of the thoracic aorta increased with age (Figure 1). The 24 M group showed significantly increased aortic media thickness compared to 2 M and 12 M groups (*P* < 0.001). To evaluate the change in profibrotic and proinflammatory proteins, western blot analyses of TGF-*β*, collagen IV, and fibronectin were performed (Figure 2). The expression of TGF-*β* significantly increased in the 24 M group compared with the 2 M (*P* = 0.001) and 12 M groups (*P* = 0.04, Figure 2(b)). The expression of collagen IV gradually increased with age (2 M versus 12 M, *P* = 0.03; 2 M versus 24 M, *P* < 0.001; 12 M versus 24 M, *P* = 0.015; Figure 2(c)). The expression of fibronectin significantly increased with age (2 M versus 12 M, *P* = 0.025; 2 M versus 24 M, *P* < 0.001; 12 M versus 24 M, *P* = 0.041; Figure 2(d)).

### 3.2. Influence of Age on Expressions of PRR, ACE, ACE2, and Ang II and Serum Levels of Ang II and Renin

**Figure 3:**
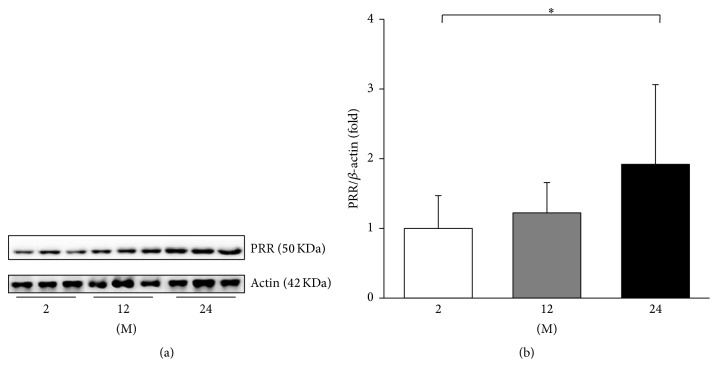
Western blot of PRR. (a) Representative western blots of PRR. (b) There was a marked increase in the expression of PRR in the 24 M group compared with the 2 M group. ^*∗*^ *P* < 0.05. Values are shown as mean ± SD.

**Figure 4:**
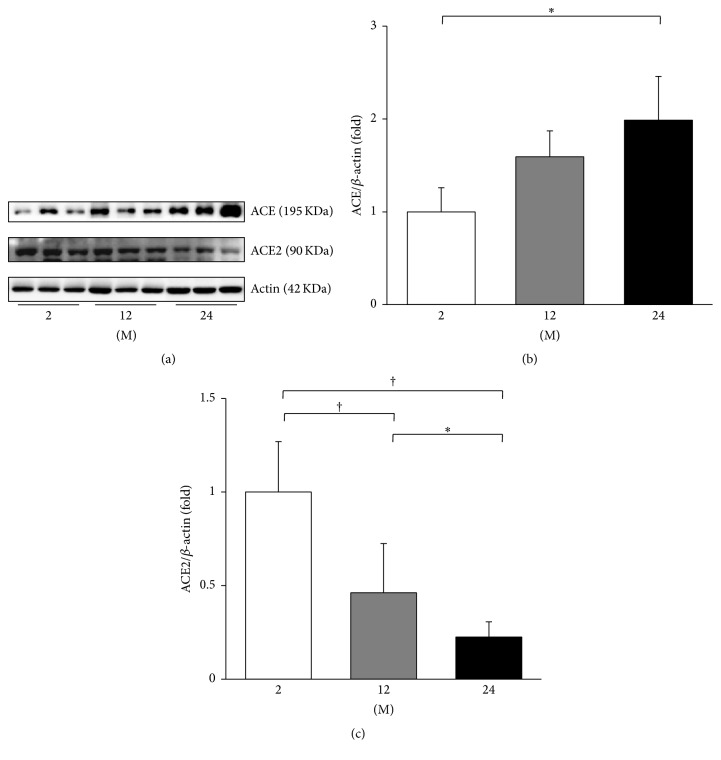
Western blots of ACE and ACE2. (a) Representative western blots of ACE and ACE2. (b) There was a marked increase in the expression of ACE in the 24 M group compared with the 2 M group. (c) The expression of ACE2 markedly decreased in the 24 M group compared with the 2 M group and 12 M group. ^*∗*^ *P* < 0.05; ^†^ *P* < 0.001. Values are shown as mean ± SD.

**Figure 5:**
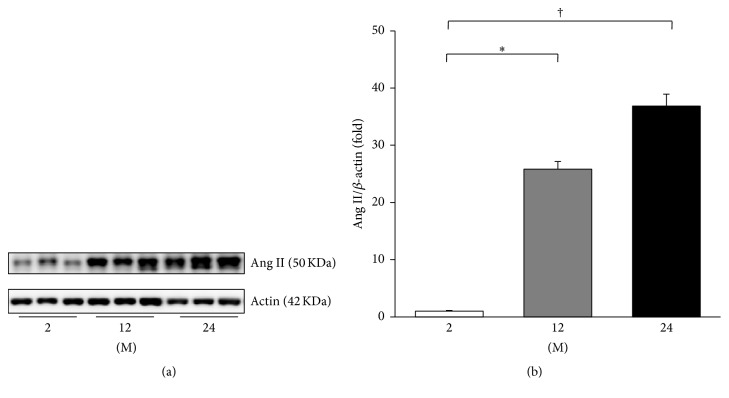
Western blot of Ang II. (a) Representative western blots of Ang II. (b) The expression of Ang II gradually increased in the 12 M group and 24 M group compared with the 2 M group. ^*∗*^ *P* < 0.01; ^†^ *P* < 0.001. Values are shown as mean ± SD.

The expressions of PRR, ACE, ACE2, and Ang II were evaluated. The expression of PRR significantly increased in the 24 M group compared with the 2 M group (*P* = 0.045, Figure 3). ACE converts Ang I to Ang II and ACE2 metabolizes Ang II; therefore the expression of these enzymes contributes to the Ang II levels [15]. Western blot analysis showed that the expression of ACE increased in the 24 M group compared to the 2 M group (*P* = 0.026, Figures 4(a) and 4(b)). In contrast, the expression of ACE2 significantly decreased with age (2 M versus 12 M, *P* < 0.001; 2 M versus 24 M, *P* < 0.001; 12 M versus 24 M, *P* = 0.037; Figures 4(a) and 4(c)).  Figure 5 shows the western blot results of Ang II. Compared with the 2 M group, the expression of Ang II gradually increased in the 12 M group (*P* = 0.001) and 24 M group (*P* < 0.001).

**Figure 6:**
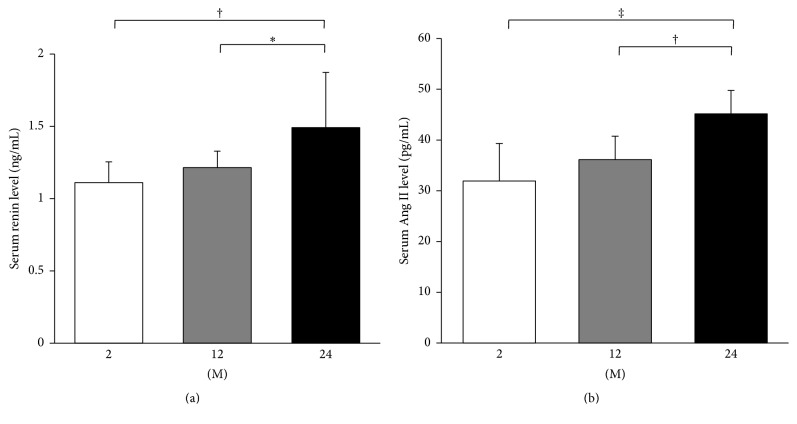
ELISA for serum levels of renin and Ang II. Serum levels of renin (a) and Ang II (b) significantly increased in the 24 M group compared to those of 2 M and 12 M groups. ^*∗*^ *P* < 0.05, ^†^ *P* < 0.01, and ^‡^ *P* < 0.001. Values are shown as mean ± SD.

Serum levels of renin and Ang II were analyzed by enzyme immunoassay. The serum renin level significantly increased in the 24 M group compared with the 2 M group (*P* = 0.005) and 12 M group (*P* = 0.034; Figure 6(a)). Serum levels of Ang II showed similar results to those of renin. The serum Ang II level significantly increased in the 24 M group compared with the 2 M group (*P* < 0.001) and 12 M group (*P* = 0.004; Figure 6(b)).

### 3.3. Influence of Age on Expressions of AT1, AT2, and Mas Receptors

**Figure 7:**
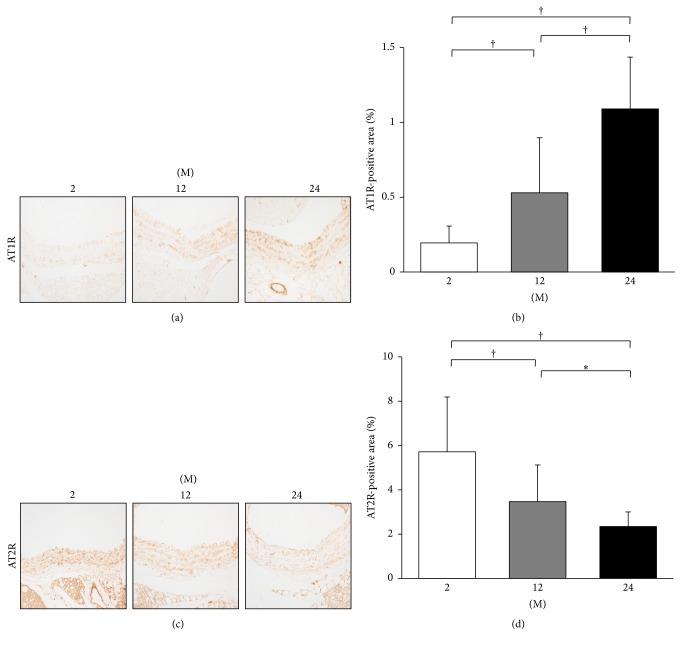
Immunohistochemistry for AT1R and AT2R. (a) Representative images of immunohistochemistry for AT1R (original magnification, 400x). (b) There was marked increase in the AT1R-positive area in the 24 M group (1.09 ± 0.35%) compared with 2 M group (0.19 ± 0.11%) and 12 M group (0.53 ± 0.37%). (c) Representative images of immunohistochemistry for AT2R (original magnification, 400x). (d) There was marked decrease in the AT2R-positive area in the 24 M group (2.34 ± 0.66%) compared with 2 M group (5.72 ± 2.47%) and 12 M group (3.47 ± 1.66%). ^*∗*^ *P* < 0.01; ^†^ *P* < 0.001. Values are shown as mean ± SD.

**Figure 8:**
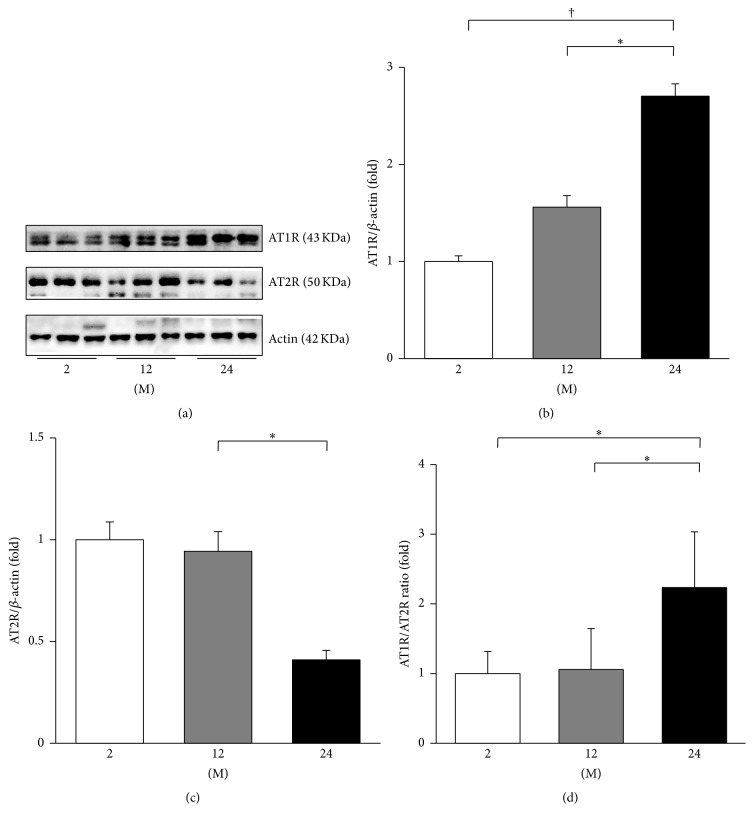
Western blots of AT1R and AT2R. (a) Representative western blots of AT1R and AT2R. (b) The expression of AT1R gradually increased in the 12 M group and 24 M group compared with the 2 M group. (c) The expression of AT2R showed a tendency of decrease in the 24 M group compared with the 2 M group, but it was not statistically significant. (d) The ratio of AT1R to AT2R significantly increased in the 24 M group compared to 2 M and 12 M groups. ^*∗*^ *P* < 0.01; ^†^ *P* < 0.001. Values are shown as mean ± SD.

Immunohistochemistry for AT1R and AT2R was performed (Figure 7). The AT1R-positive area gradually increased with age (2 M versus 12 M; 2 M versus 24 M; 12 M versus 24 M; all *P* < 0.001, Figures 7(a) and 7(b)). In contrast, the AT2R-positive area significantly decreased with age (2 M versus 12 M, *P* < 0.001; 2 M versus 24 M, *P* < 0.001; 12 M versus 24 M, *P* = 0.009, Figures 7(c) and 7(d)). Western blot analysis of AT1R and AT2R was performed (Figure 8(a)). The expression of AT1R significantly increased in the 24 M group compared with 2 M (*P* < 0.001) and 12 M groups (*P* = 0.009; Figure 8(b)). The expression of AT2R showed a tendency of decrease in the 24 M group compared with the 2 M group (*P* = 0.052; Figure 8(c)). The ratio of AT1R to AT2R significantly increased in the 24 M group compared to the 2 M (*P* = 0.001) and 12 M groups (*P* = 0.001; Figure 8(d)).

**Figure 9:**
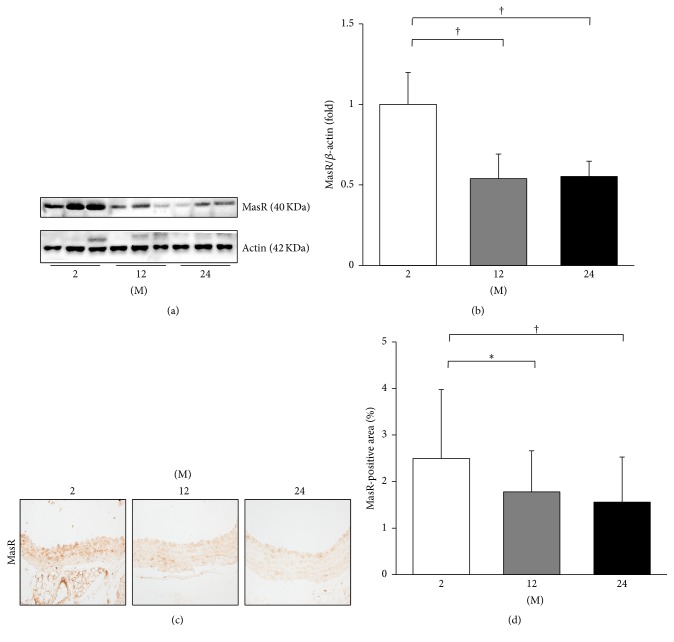
Western blot and immunohistochemistry for MasR. (a) Representative western blots of MasR. (b) The expression of MasR markedly decreased in the 12 M group and 24 M group compared with the 2 M group. (c) Representative images of immunohistochemistry for MasR (original magnification, 400x). (d) There was marked decrease in the MasR-positive area in the 24 M group (1.56 ± 0.97%) compared with 2 M group (2.50 ± 1.48%) and 12 M group (1.78 ± 0.88%). ^*∗*^ *P* < 0.05; ^†^ *P* < 0.01. Values are shown as mean ± SD.

In addition, the expression of MasR was evaluated by western blot and immunohistochemistry (Figure 9). In western blot analysis, the expression of MasR significantly decreased in the 12 M group (*P* = 0.006) and 24 M group (*P* = 0.007) compared with the 2 M group (Figures 9(a) and 9(b)). The immunohistochemistry for MasR showed similar results. The MasR-positive area significantly decreased in the 12 M group (*P* = 0.01) and 24 M group (*P* = 0.001) compared with the 2 M group (Figures 9(c) and 9(d)).

### 3.4. Influence of Age on Expressions of eNOS, Nox2, and Nox4

**Figure 10:**
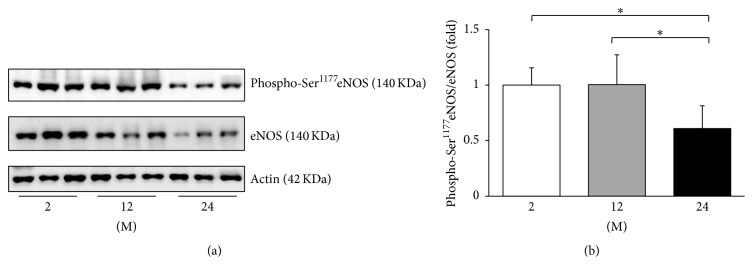
Western blots of phospho-Ser^1177^eNOS and eNOS. (a) Representative western blots of phospho-Ser^1177^eNOS and eNOS. (b) The ratio of phospho-Ser^1177^eNOS to eNOS decreased in the 24 M group compared with the 2 M and 12 M groups. ^*∗*^ *P* < 0.05. Values are shown as mean ± SD.

**Figure 11:**
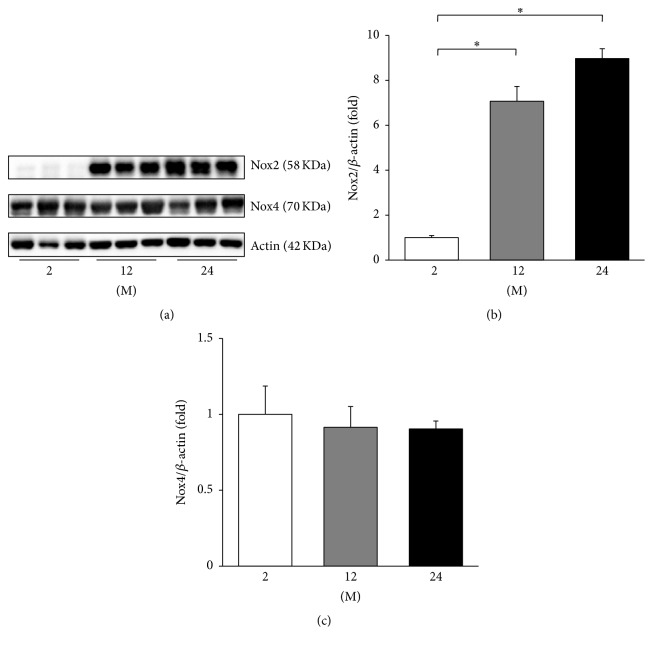
Western blots of Nox2 and Nox4. (a) Representative western blots of Nox2 and Nox4. (b) The expression of Nox2 markedly increased in the 12 M group and 24 M group compared with the 2 M group. (c) There was no significant difference in the expression of Nox4 between groups. ^*∗*^ *P* < 0.001. Values are shown as mean ± SD.

As the change in the eNOS transcription may be involved in age-related endothelial dysfunction [17], western blot analyses of phosphorylated serine 1177 (phospho-Ser^1177^) eNOS and eNOS were performed (Figure 10). The ratio of phospho-Ser^1177^eNOS to eNOS decreased in the 24 M group compared with the 2 M and 12 M groups (*P* = 0.019 and *P* = 0.018, resp.). Western blot analyses of Nox2 and Nox4 were also performed (Figure 11(a)), since the NADPH oxidases of the Nox family are known to be involved in maintaining the balance between oxidative and antioxidative systems [18]. The expression of Nox2 significantly increased in the 12 M group and 24 M group compared with the 2 M group (*P* < 0.001, Figure 11(b)). However, there was no significant difference in the expression of Nox4 between groups (Figure 11(c)).

### 3.5. Influence of Age on Expressions of SOD1 and SOD2 and Oxidative Stress

**Figure 12:**
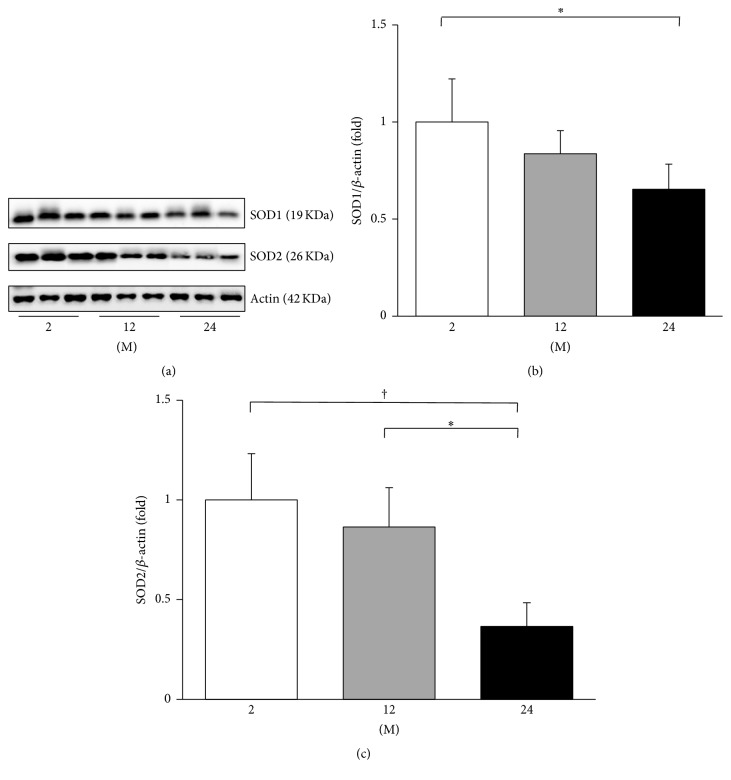
Western blots of SOD1 and SOD2. (a) Representative western blots of SOD1 and SOD2. (b) The expression of SOD1 markedly decreased in the 24 M group compared with the 2 M group. (c) The expression of SOD2 decreased in the 24 M group compared with the 2 M and 12 M groups. ^*∗*^ *P* < 0.01; ^†^ *P* < 0.001. Values are shown as mean ± SD.

The changes in the antioxidant proteins SOD1 and SOD2 were performed by western blot analysis (Figure 12(a)). The expression of SOD1 decreased in the 24 M group compared with the 2 M group (*P* = 0.007, Figure 11(b)). The expression of SOD2 also decreased in the 24 M group compared with the 2 M and 12 M groups (*P* < 0.001 and *P* = 0.001, resp., Figure 12(c)).

**Figure 13:**
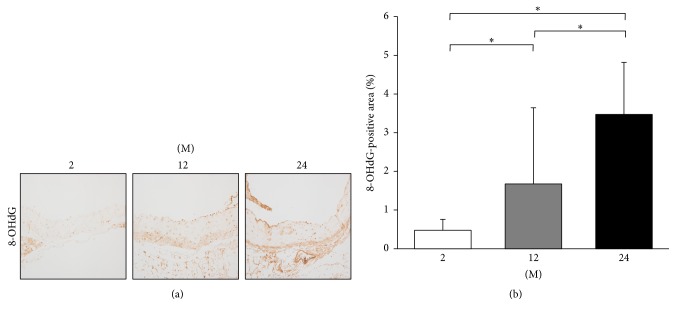
Immunohistochemistry for 8-OHdG. (a) Representative images of immunohistochemistry for 8-OHdG (original magnification, 400x). (b) There was marked increase in the 8-OHdG-positive area in the 24 M group (3.47 ± 1.34%) compared with 2 M group (0.48 ± 0.28%) and 12 M group (1.67 ± 1.97%). ^*∗*^ *P* < 0.001. Values are shown as mean ± SD.

**Figure 14:**
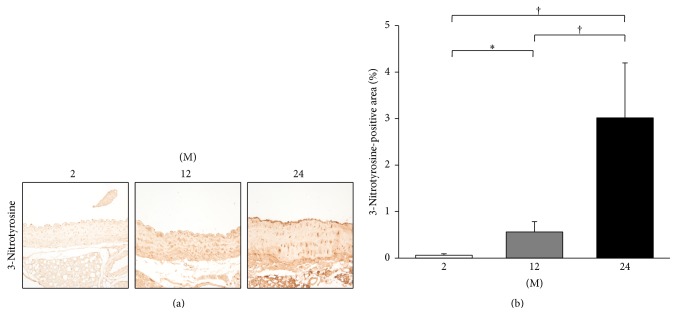
Immunohistochemistry for 3-nitrotyrosine. (a) Representative images of immunohistochemistry for 3-nitrotyrosine (original magnification, 400x). (b) There was marked increase in the 3-nitrotyrosine-positive area in the 24 M group (3.02 ± 1.18%) compared with 2 M group (0.06 ± 0.03%) and 12 M group (0.56 ± 0.22%). ^*∗*^ *P* < 0.05; ^†^ *P* < 0.001. Values are shown as mean ± SD.

Immunohistochemistry for 8-OHdG and 3-nitrotyrosine was done for assessment of oxidative stress. Immunohistochemistry for 8-OHdG showed that the 8-OHdG-positive area gradually increased with age (2 M versus 12 M; 2 M versus 24 M; 12 M versus 24 M; all *P* < 0.001; Figure 13). Immunohistochemistry for 3-nitrotyrosine showed that the 3-nitrotyrosine-positive area significantly increased with age (2 M versus 12 M, *P* = 0.029; 2 M versus 24 M, *P* < 0.001; 12 M versus 24 M, *P* < 0.001; Figure 14).

## 4. Discussion

The novel finding of the present study is that the thoracic aorta of aging mice exhibits altered RAS components which are characterized by an enhanced PRR-ACE-Ang II-AT1R axis and a reduced ACE2-MasR axis. The altered expression of RAS components was associated with fibrosis, inflammation, and oxidative stress in the aging aorta.

The activation of tissue-specific RAS components has been demonstrated in various tissues. It is well known that the intrarenal RAS activation is associated with the renal aging process [4]. Age-associated cardiac remodeling involves the myocardial RAS activation [19], and the skeletal RAS is involved in the age-related osteoporosis of mice [20]. There also is evidence that RAS blockade ameliorates cognitive decline and neurodegenerative disorders associated with aging [21]. Ang II is the key peptide in the organ damage associated with RAS activation [5]. Our results showed consistent findings with previous literature. The aortic expressions of ACE, Ang II, and AT1R increased in 24-month-old mice, whereas those of ACE2 and MasR decreased. These findings not also support the classical concept of RAS activation, the upregulation of ACE-Ang II-AT1R axis, in arterial aging, but also show that the antagonizing ACE2-MasR axis is downregulated. In addition, the expression of PRR was significantly increased in 24-month-old mice. It was shown that binding of prorenin or renin to the PRR induced mesangial cells to produce TGF-*β* and to increase fibronectin and collagen synthesis independently of Ang II [22]. Recently, PRR was shown to be essential for cell survival and downregulation of vascular inflammation in murine vascular smooth muscle cells [23]. Moreover, our results showed that the serum levels of renin and Ang II significantly increased with age, which implicates the activation of humoral RAS. These findings altogether implicate that the arterial aging is associated with the imbalance between various RAS components.

TGF-*β* is one of the most important downstream events of the RAS and Ang II is implicated in arterial aging [2]. It was reported that aortic TGF-*β* accumulation may be related to the age-associated increase in aortic fibronectin and collagen [24, 25] and that the expression of TGF-*β* and fibronectin are both regulated by Ang II [26]. Consistently, our results demonstrated that the aortic media thickness and the expression of TGF-*β*, collagen IV, and fibronectin increased in 24-month-old mice compared with 2-month-old and 12-month-old mice.

It is known that the expression of the AT2R is ubiquitous in fetal tissues of rodents and it decreases after birth, remaining at a low level in adulthood in adrenal medulla, uterus, ovary, vascular endothelium, and distinct brain areas [27]. In this study, the western blot analysis showed that the expression of AT1R and the ratio of AT1R to AT2R increased with age. However, the expression of AT2R did not change significantly, and the expression of AT2R was similar between 2-month-old mice and 12-month-old mice. Similarly, Gao et al. reported that adult mice (10–14 week-old) exhibited significantly higher AT2R expression in total protein extracts compared to fetuses and neonates in neural tissues, heart, lung, liver and kidney [28]. They speculated that the difference in the protein measurement method may cause this discrepancy. The conventional concept on AT2R expression profiles before and after birth was derived from autoradiographic data [29–31], ligand binding experiments [32–34], and* in situ* hybridization [35–37]. Autoradiography and ligand binding primarily determine mature receptors only on the cell surface.* In situ* hybridization detects mRNA but is not a reliable technique to quantify gene expression. By contrast, western blot measures total AT2R protein including both plasma membrane (mature) and cytoplasmic (immature) protein. In addition, different regions, even in the same organ, may exhibit different AT2R expression level. These findings may support our results in which the total AT2R protein expression did not decrease in 12-month-old mice compared with 2-month-old mice.

It is well known that oxidative stress and impaired antioxidant defense mechanisms are mainly involved in the vascular aging process [38]. Ang II activates intracellular NADPH oxidase via the AT1R to generate superoxide anion (O_2_^−c^). Excessive superoxide production promotes the uncoupling of eNOS, which in turn reduces nitric oxide availability and enhances ROS production [39, 40]. In this study, the 24-month-old mice exhibited decreased aortic expression of Nox2 and phospho-Ser^1177^eNOS expression, both of which might have enhanced the ROS production. In resting endothelial cells, serine 1177 (Ser1177) is usually not phosphorylated. Phosphorylation is induced when the cells are exposed to oestrogens, vascular endothelial growth factor, insulin, bradykinin, or fluid shear stress. Phosphorylation of the Ser1177 residue increases the enzyme activity [41]. Thus our results suggest that the eNOS is activated with age and thus results in oxidative stress. In our study, the phospho-Ser^1177^eNOS expression was nearly similar between 12-month-old mice and 24-month-old mice. Zecchin et al. previously reported a similar finding, which showed that phospho-Ser^1177^eNOS expression was similar in thoracic aorta of 2-month-old and 12-month-old Wistar rats [42]. This may be because the eNOS enzyme system is overactivated in aged blood vessels as a compensatory mechanism to counterbalance endothelial dysfunction induced by age-associated oxidative stress [43].

Ang II increases ROS formation also in the mitochondria level [44]. Consistently, our results showed that the aortic expressions of mitochondrial antioxidant enzymes, SOD1 and SOD2, decreased in 24-month-old mice and that the 8-OHdG-positive area, an oxidative stress marker, increased with age. The 3-nitrotyrosine-positive area, a marker for inflammation and nitric oxide production, also increased with age. However, there was no significant change in the aortic expression of Nox4. Nox2 is known to promote the development of endothelial dysfunction, hypertension, and inflammation [45]. Unlike Nox2 that primarily produce superoxide, Nox4 has been shown to produce hydrogen peroxide (H_2_O_2_) [46], which cannot scavenge nitric oxide [47]. Recent evidence suggest that Nox4 may have a protective role in the vasculature during stress [45]. An* in vivo* study demonstrated that endothelial-specific overexpression of Nox4 enhanced endothelial function and reduced blood pressure [48]. In Nox4^−/−^ mice, endogenous Nox4 protected the vasculature during ischemic or inflammatory stress [49]. In addition, Nox4 expression was decreased in human abdominal aortic aneurysm in spite of increased oxidative stress [50]. In this study, although it was not significant, there was a decreasing tendency in aortic expression of Nox4. We speculate that the role of Nox4 may not be crucial in the arterial aging.

In conclusion, our results demonstrate that arterial aging is associated with enhanced PRR-ACE-Ang II-AT1R axis and reduced ACE2-MasR axis, which results in fibrosis, inflammation, and oxidative stress of the thoracic aorta. The altered expression of RAS components may explain the increased susceptibility to vascular injury and cardiovascular disease in the elderly population. Targeting these signaling molecules may provide specific therapeutic strategies for cardiovascular disease to this population.
